# An MRI-compatible defibrillator: initial testing in volunteers and swine

**DOI:** 10.1186/1532-429X-18-S1-O122

**Published:** 2016-01-27

**Authors:** Ehud J Schmidt, Ronald D Watkins, Menekhem M Zviman, Michael Guttman, Wei Wang, Henry Halperin

**Affiliations:** 1 grid.62560.370000000403788294Brigham and Womens Hospital, Boston, MA USA; 2 Radiology, Stanford, Stanford, CA USA; 3 Cardiology, Johns Hopkins, Baltimore, MD USA

## Background

Performing MRI-guided interventions, such as for the treatment of ventricular tachycardia, requires the ability to rapidly detect and treat acute cardiac conditions, should they occur in the MRI. We developed MRI-compatible 12-lead ECG systems, and demonstrated detection of acute ischemia during imaging [Tse, MRM. '13,Zhang, MRM. '15 ], providing a tool to non-invasively detect cardiac events in the MRI. It is presently necessary to remove patients from the MRI to treat cardiac events. This increases risk, consequently restricting MRI imaging, as well as MRI-guided intervention, to lower-risk patients and lower-risk procedures. Defibrillation in the bore could, therefore, accelerate the growth of diagnostic (such as exercise stress-perfusion and acute post-infarct studies) and therapeutic MRI studies. The study objective is to develop a defibrillation system that can safely be used on patients inside a 1.5 Tesla MRI. In addition, this system should not compromise MRI image quality.

## Methods

**Figure 1:**
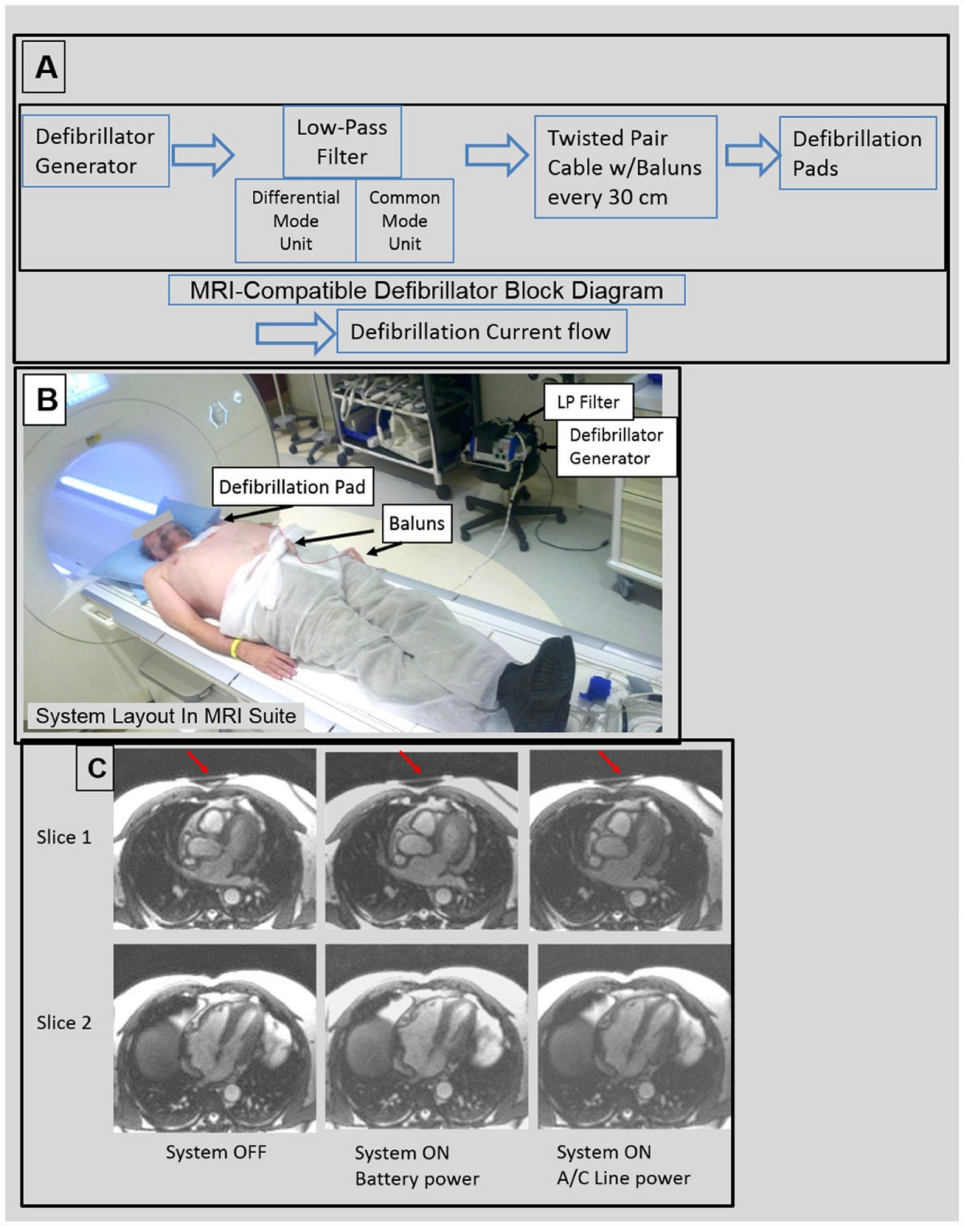
**(A) Block diagram of MRI-compatible defibrillator**. 7-pole Low-pass filter has diffential-mode and common-mode units to maximally suppress unwanted RF current induction into MRI. (B) The layout of the system inside the MRI room. (C) 1.5T MRI SSFP images with defibrillator generator OFF, generator ON powered from the battery, and generator ON powered from the line (worst SNR condition). Red arrows denote location of defibrillation pads.

The output of a commercial Zoll Medical (Chelmsford, MA) M-series defibrillation generator (Figure 1A) was connected to a novel 7-pole low-pass filter, designed to reduce by 80 dB emission of both differential-mode and common-mode 63.8 MHz signals into the scanner, while maintaining the essential 67-KHz defibrillator-pad ON/OFF detection utility of the generator, and its ECG monitoring utility. The low-pass filter was connected to commercial Zoll multifunction defibrillator pads on a subject's chest via 3-meter-long high-voltage twisted-pair cables, mounted with tuned RF-traps ("Baluns") at 30-cm increments (Figure 1B). Volunteer cardiac SSFP imaging was performed on a Siemens 1.5T Aera using the scanner's cardiac array, with the generator in the MRI suite, but outside the 5-Gauss line. Image quality was compared under three conditions; with (a) the generator OFF, (b) generator ON in ECG-monitoring mode, powered from the on-board battery, or (c) the A/C power outlet. In addition, multiple full-power (2000V) defibrillations were performed on a swine, inside the MRI, using the system.

## Results

A slight 2 and 1 dB reduction in image SNR was observed (Figure 1C) with the system running on A/C power, and running on battery, respectively, relative to the system OFF. The system OFF state displayed no discernable SNR penalty relative to removal of the system from the room. Image artifacts from the defibrillator pads extended only ~6 mm below the skin. Defibrillator-pad heating did not occur. The swine defibrillations were completed without any complications.

## Conclusions

Defibrillation inside the MRI may be performed with a modified commercial defibrillator system, enabling the performance of higher-risk imaging and MRI-guided interventions.

